# Adhesion of *Bacteroides vulgatus* and *Fusobacterium varium* to the Colonic Mucosa of Healthy Beagles

**DOI:** 10.3390/vetsci11070319

**Published:** 2024-07-16

**Authors:** Mohsen Hanifeh, Mirja Huhtinen, Yannes S. Sclivagnotis, Ulrike Lyhs, Thomas Grönthal, Thomas Spillmann

**Affiliations:** 1 Department of Equine and Small Animal Medicine, Faculty of Veterinary Medicine, University of Helsinki, Viikintie 49, 00014 Helsinki, Finland; thomas.gronthal@helsinki.fi (T.G.); thomas.spillmann@helsinki.fi (T.S.); 2 Orion Corporation, Orion Pharma, R&D, Orionintie 1A, 02200 Espoo, Finland; mirja.huhtinen@orionpharma.com (M.H.); yannes.sclivagnotissiotkas@orion.fi (Y.S.S.); ulrike.lyhs@orionpharma.fi (U.L.)

**Keywords:** anaerobic bacteria, bacterial adhesion, *Bacteroides vulgatus*, *Fusobacterium varium*, dogs, colonic mucosa

## Abstract

**Simple Summary:**

The presence of Bacteroidetes and Fusobacteria phyla are reported to decrease in dogs with chronic enteropathies, likely due to increased oxidative stress in the colon’s inflammatory environment. Adherence to the colonic mucosa is considered an essential step for these bacteria to colonize and interact with the host’s epithelium and immune system. No studies on dogs have investigated the adhesion of *Bacteroides vulgatus* and *Fusobacterium varium* on paraffin-embedded canine colonic mucosa. This study aims to examine the adhesion capacities of these bacterial species to paraffin-embedded colonic mucosa from healthy dogs and investigate their hydrophobicity properties to determine if this factor explains differences in adhesion capability. The results indicated that both canine *B. vulgatus* and *F. varium* adhered in high numbers to canine colonic mucosa; however, *B. vulgatus* had higher hydrophobicity but adhered in lower numbers than *F. varium*. In conclusion, both bacteria have probiotic potential, but further research is necessary to determine the efficacy and safety of the strains to be used, and other factors influencing attachment beyond hydrophobicity.

**Abstract:**

The relative abundances of Bacteroidetes and Fusobacteria phyla have been reported to be decreased in dogs with chronic enteropathies. In colitis, obligate anaerobes (e.g., *Bacteroides* and *Fusobacterium*) are likely to vanish in response to the heightened oxidative stress in the colon’s inflammatory environment. The ability to adhere to the colonic mucosa is viewed as an essential step for obligate anaerobic bacteria to colonize and subsequently interact with the host’s epithelium and immune system. The reintroduction of a balanced community of obligate anaerobic bacteria using probiotics can restore the microbial function in the intestine. We found no studies on dogs regarding the adhesion properties of *Bacteriodes vulgatus* and *Fusobacterium varium* on paraffin-embedded canine colonic mucosa. Thus, the objective of this study is to investigate the adhesion capacities of these two bacterial species to paraffin-embedded colonic mucosa from healthy dogs. Additionally, we investigated their hydrophobicity properties to determine whether differences in adhesion capability can be explained by this factor. The results of our study showed that *B. vulgatus* adhered significantly lower than *F. varium* to the canine colonic mucosa (*p* = 0.002); however, *B. vulgatus* showed higher hydrophobicity (46.1%) than *F. varium* (12.6%). In conclusion, both bacteria have potential as probiotics, but further studies will be required to determine the efficacy and safety of the strains to be used, which strains to use, and the reasons other than hydrophobicity for attachment.

## 1. Introduction

The gastrointestinal tract of dogs is densely colonized by a heterogeneous group of microorganisms known as the gastrointestinal microbiota. In healthy dogs, the fecal microbiome is co-dominated by three phyla: Bacteroidetes, Fusobacteria, and Bacillota (synonym Firmicutes) [1]. The 16S rRNA sequences generated from the colon luminal content of healthy dogs revealed that about 40% of sequences represents Bacillota, and Bacteroidetes and Fusobacteria both represented nearly 30% of sequences each [2]. Alterations in the composition and function of the gut microbiota, or dysbiosis, have been reported in dogs with chronic enteropathies (CE), and in human patients suffering from inflammatory bowel diseases (IBDs) [1,3]. Canine CE is characterized by persistent or recurrent gastrointestinal symptoms, such as vomiting, diarrhea, weight loss, and abdominal pain [4]. CE is currently classified based on clinical response to sequential treatment trials as food-responsive enteropathy (FRE), antibiotic-responsive enteropathy (ARE), immunosuppressant-responsive enteropathy (IRE), and non-responsive or refractory enteropathies (NREs) [4]. The distal part of the normal intestine is characterized by a low level of oxygen and by the presence of a large number of obligate anaerobic bacterial communities [3]. Compared to facultative microbes, anaerobic bacteria are more likely to disappear in an inflammatory environment because they have a lower capacity for dealing with oxidative stress [5]. In humans with IBD, studies have reported a decline in obligate anaerobes belonging to the phylum Bacillota and an increase in facultative anaerobes, including members of the family *Enterobacteriaceae* [5]. In dogs with steroid-responsive enteropathies (SREs), a subtype of CE, a reduction in the relative abundance of Bacteroidetes and Fusobacteria phyla has been observed [6]. In another study, the relative abundances of genera *Bacteroides* and *Fusobacterium* were decreased in Yorkshire Terriers with IBD compared to healthy control dogs [7].

*Bacteroides* species are Gram-negative, obligate anaerobic, bile-resistant, non-spore-forming bacteria [8]. They produce short-chain fatty acids (SCFAs), which are the main energy source for colonocytes, and they are decreased in dogs with CE [9]. *Bacteroides* species were found to decrease not only at baseline, but also at 3 and 8 weeks from the beginning of prednisone treatment in dogs with SREs. They eventually reached abundances comparable to the healthy controls after one year from prednisone treatment [6].

The genus *Fusobacterium* includes several species of obligatory anaerobic bacteria which have been identified as one of the principal microbial players of the canine gut microbiota [2,10,11]. *Fusobacterium* species are able to degrade proteins to obtain their preferred growth substrates such as amino acids and peptides [12]. These abilities are probably the reason for high abundance of *Fusobacterium* in carnivorous animals [12]. For example, *Fusobacterium* (F.) *varium* exerts not only anti-inflammatory effects but also sustains enterocytes by producing butyrate from protein fermentation and acts as a potent antagonist of pathogen colonization [13]. Although *F. varium* has been linked with ulcerative colitis in humans [14], this negative association has not been observed in dogs [11]. In dogs with SREs, the relative abundance of *Fusobacterium* was severely decreased and remained significantly low after 8 weeks of prednisone treatment. However, the abundance of *Fusobacterium* was no longer significantly different from healthy controls after one year from the treatment [6].

The reintroduction of a balanced community of obligate anaerobic bacteria using fecal microbiota transplantation (FMT) [15] or manufactured multi-species gut colonizers [16] can restore the microbial function in the intestine of humans. In dogs with CE, FMT was reported to result in a higher diversity of the fecal microbiome and an increase in the proportion of Fusobacteria [17], and in dogs with poorly responsive CE, FMT was suggested to be used as an adjunctive therapy [18]. The application of VSL#3^®^ or VivoMixx^®^ (BIOSCEM, Bucharest, Romania) as multi-strain probiotics in dogs with IBD also showed beneficial effects on mucosal homeostasis and dysbiosis [19,20].

Adherence to the intestinal epithelium and mucus is considered an important step for intestinal bacteria to colonize and further interact with the host’s epithelium and immune system [21,22]. The intestinal tract of humans and animals contains trillions of bacteria which possess adhesin molecules such as FimH, Ace, and FadA allowing the bacteria to bind to various surfaces of the host [23,24,25]. The ability of binding to the host gastrointestinal (GI) mucosa is one of the main criteria in probiotic strain selection [26,27].

Recently, we demonstrated the feasibility of a method to investigate host–microbiome interactions using paraffin-embedded intestinal tissue samples of healthy Beagles [28]. We successfully investigated the adhesive capacity of *Enterococcus faecalis* and *Enterococcus faecium* on paraffin-embedded duodenal tissue samples in dogs [28]. Cellular organization and structures are more physiological in paraffin-embedded tissue sections than immobilized mucus or isolated intestinal epithelial cells [22,29,30,31,32,33]. Due to the archiving of paraffin-embedded biopsy samples, this method offers an opportunity for using these readily available resources in bacterial adhesion studies under different disease conditions (e.g., intestinal inflammation, tumors, etc.), reducing the need to collect new samples from different patient groups and therefore speeding up research time.

After a systematic search, we found no study in dogs investigating the adhesion of bacteria of the two major phyla of the dog microbiome, Bacteroidetes and Fusobacteria, to paraffin-embedded canine intestinal mucosa. Therefore, in this study, we aimed to investigate the adhesion capacities of two representatives of Bacteroidetes and Fusobacteria to paraffin-embedded colonic mucosa of healthy dogs, namely of canine *Bacteriodes (B.) vulgatus* and of a *Fusobacterium* isolate closely related to *Fusobacterium varium* (>97% sequence similarity based on 16S rRNA gene sequences). Since bacterial cell surface hydrophobicity is an important nonspecific factor for higher bacterial adhesion, we investigated their hydrophobicity properties to determine the association of bacterial adhesion with hydrophobicity [34].

## 2. Materials and Methods

### 2.1. Bacteria and Growth Conditions

A list of used bacterial strains in this study is provided in Table 1. Canine *B. vulgatus* and *F. varium* were isolated from feces samples retrieved from a colony of 36 Beagle dogs with an age of 1.5–3.5 years and a body weight of 7–17 kg. The dogs were considered healthy based on their history, physical examination, and quality of feces. They had not received any antibiotics and were vaccinated once/year and dewormed with fenbendazole twice/year. The bacteria were first cultured in fastidious anaerobe agar (FAA) with horse blood (Thermo Scientific ™ PB0225A, Waltham, MA, USA) for 48 h at +37 °C under anaerobic conditions created by a sealed anaerobic bag and AnaeroGen^TM^ Compact kit (Oxoid, Thermo Scientific, Basingstoke, Hampshire, UK). Then, the bacterial colonies were inoculated into fastidious anaerobe broth (FAB) (T037, Tammer Biolab, Tampere, Finland) and cultivated for 5 days at +37 °C under anaerobic conditions created by a sealed anaerobic jar and AnaeroGen^TM^ 2.5L kit (Oxoid, Thermo Scientific, Basingstoke, Hampshire, UK).

### 2.2. Colonic Tissue Samples

For this study, paraffin-embedded tissue samples from colon of six healthy laboratory Beagle dogs were used. The Beagles were six-year-old intact females with a median body weight of 12.5 kg (range 10.2–14.2 kg). They were fed a standard commercial diet and were considered healthy based on their history, clinical exam, complete blood count, serum chemistry panel, and histological and fecal examinations. Colonic tissue samples from the dogs were collected immediately after post-mortem examinations of an unrelated study approved by the Finnish National Animal Experiment Board (ethical license ESAVI/7290/04.10.03/2012). The collected samples were immediately fixed in buffered 4% formaldehyde, embedded in paraffin, sectioned by rotary microtome at thickness of 3–5 µm, and mounted on glass slides. The Beagle dogs were euthanized after finalizing an unrelated, ethically approved study assessing adverse effects of intra-articular botulinum toxin A.

### 2.3. Collecting and Labeling Bacteria

Collecting and staining of cultured *B. vulgatus* and *F. varium* were performed as described previously [28]. Briefly, after centrifugation of cultured *B. vulgatus* and *F. varium*, the collected bacteria were washed twice with 0.1 M sodium bicarbonate buffer (pH 8.3) and finally were adjusted to the OD_600_ value of 1.0 with the same buffer.

To stain the bacteria, Alexa Fluor 488 NHS Ester (A20000, Thermo Fisher Scientific) was used. Preparation of Alexa Fluor 488 (AF488) and staining of bacterial cells were performed as described previously [28,35]. Briefly, we added 0.5 mL of bacterial suspensions to tubes containing AF488 (20 µg). Then, the tubes were rotated for 1 h at room temperature (RT) with end-over-end rotation. The stained cells were collected by centrifugation (16 000 g for 5 min) and were washed thrice with tris-buffered saline (TBS), pH 7.4. To minimize nonspecific binding of bacterial cells to the colonic epithelium, the collected bacterial cells were re-suspended in 0.5 mL of blocking solution (12% fetal bovine serum, 2% bovine serum albumin, 0.2% Triton X-100 in TBS containing 0.001% Tween 20). OD600 values of the labeled bacterial suspensions were checked, re-adjusted to OD600 = 1, and also diluted to OD600 = 0.5 in blocking solution. All the procedures after adding AF488 stain were kept protected from light.

### 2.4. Bacterial Adhesion to Colonic Mucosa

We used the adhesion assay as described by Isaacson et al. (2018) with slight modifications that we applied in our previous study [28]. Briefly, after deparaffinization of paraffin-embedded colonic tissue slides, the slides were blocked by adding blocking solution incubating them at RT for 6 h. Then, the blocking solution was discarded and 0.5 mL of the labeled bacterial suspensions (OD_600_ = 0.5 and 1) were gently placed on slides and incubated in a dark moist chamber overnight at +4 °C. Due to the long incubation time, an overnight incubation temperature of +4 °C was chosen to protect the biological material from deterioration and to allow the bacteria to bind to the mucosa. The slides were then washed three times with PBS, stained with nucleic acid-binding dye 4,6’-diamidino-2-phenylindole (DAPI) (1 µg/mL in PBS) for 15 min at RT and washed again in PBS. Then, the slides were mounted and the adherent bacteria were examined by epifluorescence microscopy (Leica DM 4000B, Wetzlar, Germany). Epifluorescence I3 filter and DAPI filter were used for detecting mucosa-attached fluorescent bacteria and identification of host–cell nuclei, respectively. To show the structure of the colonic mucosa and its attached fluorescent bacteria at the same time, we used a combination of epifluorescence I3 and a phase contrast filter. In order to estimate the adhesion of each strain, the number of mucosal attached bacteria was counted in 20 random microscopic fields (3.5 × 10^4^ μm^2^ each) through three replicate experiments. The results of attached bacteria were reported as a mean of three replicates.

To control unspecific binding, 20 µL of blocking solution was added to each well of a diagnostic slide (Waldemar Knittel Glasbearbeitungs GmbH, Braunschweig, Germany) and incubated in a moist chamber overnight at RT. The following day, 10 µL of each bacterial suspension was loaded to two parallel wells in the diagnostic slides while adding stained bacterial suspensions to tissue slides. These slides then were treated identically to the tissue slides.

### 2.5. Bacterial Hydrophobicity

Hydrophobicity of *B. vulgatus* and *F. varium* was determined by detecting the bacterial adhesion to hydrocarbons as described previously [28]. Briefly, cultured *B. vulgatus* and *F. varium* were harvested by centrifugation (5000× *g*, +4 °C, 15 min); then, the collected cells were washed two times with PBS (pH 7.0) and re-suspended in the same solution to an optical density (OD600) of 0.525–0.584 (A0). Then, in a 1:3 proportion, xylene (Bio-Optica, Milan, Italy) was added to the bacterial cell suspension in a glass tube and vortexed rigorously for 2 min and incubated for 2 h at RT. The aqueous phase was removed, and the OD600 nm was determined (A1) and compared with the initial value. The hydrophobicity percentage (%H) was calculated using the equation %H = [(A0 − A1)/A0] × 100 and was expressed as follows: 0–35%, low hydrophobicity; 36–70%, medium hydrophobicity; and 71–100%, high hydrophobicity. All measurements were made in quadruplicate and the mean hydrophobicity percentage was reported.

### 2.6. Statistical Analyses

Shapiro–Wilk test was used to check the normality of data distribution. Since the data were non-normally distributed, nonparametric tests were used for statistical analysis. The differences between the numbers of adhered canine *B. vulgatus* (OD 0.5 and 1) and *F. varium* (OD 0.5 and 1) cells to the colonic tissue samples were assessed using the Mann–Whitney *U* non-parametric test. The data are presented as median (range). For all analyses, we considered values of *p* < 0.05 as significant. All statistical analyses were performed using the GraphPad Prism 9.0.0 (GraphPad Software, Inc., San Diego, CA, USA).

## 3. Results

### 3.1. Adherence of B. vulgatus and F. varium to Canine Colonic Mucosa

**Figure 1:**
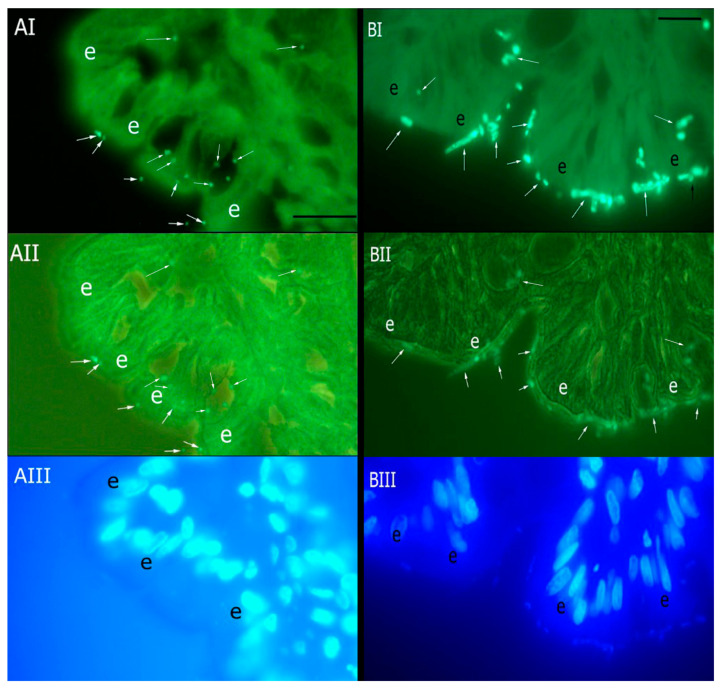
Attachment AF488-stained *B. vulgatus* DF28 (**A**) and *F. varium* DF30 (**B**) to tissue sections of the colonic mucosa of Beagles. In this figure, captured images by epifluorescence (**I**), epifluorescence and phase contrast combination (**II**), and DAPI (**III**) filters are illustrated. Symbol “e” indicates the epithelial layer of the colonic mucosa, and arrows show attached bacteria to the epithelium and lamina propria. Scale bars, 50 µm.

Canine *B. vulgatus* DF28 and *F. varium* DF30 strains were stained with AF488 and loaded on paraffin-embedded colonic tissue sections of healthy Beagles (Figure 1). *B. vulgatus* DF28 and *F. varium* DF30 attached to the different parts of the mucosa including epithelial layer and lamina propria.

### 3.2. Mucosal Adhesion of B. vulgatus and F. varium

**Figure 2:**
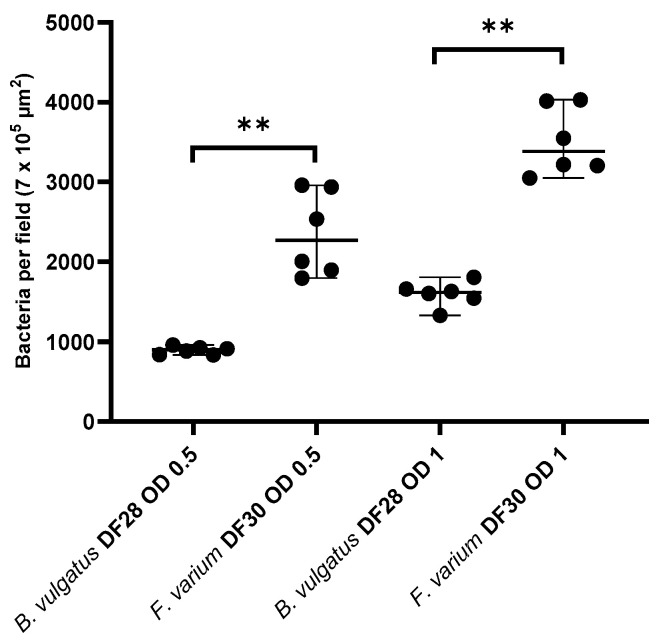
Adherence of canine *B. vulgatus* and *F. varium* to the colonic mucosa of healthy Beagles has been displayed by scatter plot. Quantity of attached bacteria is presented as median (range). OD, optical density at 600 nm; ** *p* > 0.01.

The number of *B. vulgatus* DF28 and *F. varium* DF30 adhered to the colonic mucosa of healthy Beagles is shown in Figure 2. The results showed that *B. vulgatus* DF28 adhered significantly lower than *F. varium* DF30 to the canine colonic mucosa at both OD_600_ = 0.5 (899 [834–958] vs. 2271 [1796–2959] bacteria per field; *p* = 0.002) and OD_600_ = 1 (1617 [1329–1805] vs. 3383 [3049–4029] bacteria per field; *p* = 0.002).

### 3.3. Hydrophobicity of B. vulgatus and F. varium

**Figure 3:**
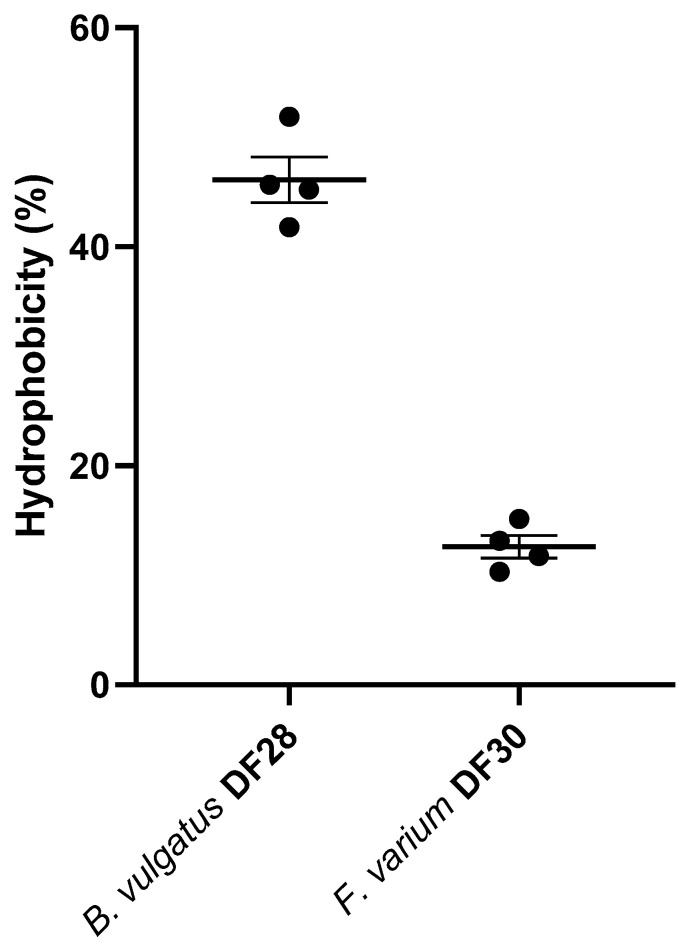
Hydrophobicity (%) of canine *B. vulgatus* DF28 and *F. varium* DF30. The graph shows hydrophobicity percentage from four repetitions with their mean values ± SEM.

The results showed that canine *B. vulgatus* DF28 had higher (46.1%, medium level) hydrophobicity properties than canine *F. varium* DF30 (12.6%) (Figure 3).

## 4. Discussion

The ability of commensal or pathogenic bacteria to adhere to the intestinal mucosa of the host allows them to avoid clearance by mucosal secretions and peristalsis [36]. This ability is one of the main criteria in the selection of probiotic strains [26,27]. In this study, for the first time, we investigated the adhesion properties of canine *B. vulgatus* and *F. varium* strains to the paraffin-embedded colonic tissue samples of healthy Beagles and its possible relation to the hydrophobicity of bacteria.

In our study, *B. vulgatus* DF28 adhered to the colonic mucosa with median of 899 bacteria per field in OD_600_ = 0.5 and 1617 in OD_600_ = 1. Nakano et al. (2008) showed that the adherence of *B. vulgatus* to HEp-2 cell lines was 75% (6/8) for isolates from children with diarrhea and 83.3% (5/6) for isolates from children without diarrhea [37]. Another study investigated the adherence of *B. vulgatus* strains isolated from the large intestinal mucosa of ulcerative colitis (UC) patients and non-UC individuals to tissue-cultured cells [38]. The adherence to tissue-cultured cells was higher in UC-derived strains (36.5 +/− 7.9%) than in non-UC-derived strains (13.2 +/− 7.7%). *B. vulgatus* strains contain a variety of cell surface molecules such as adhesins, outer membrane proteins, and a polysaccharide capsule that are either critical or advantageous for colonization [37,39].

Our research revealed that *F. varium* DF30 displayed a significant higher adherence to the colonic mucosa compared to *B. vulgatus* DF28. Making chains with multiple spindle-shaped rods and attaching to the mucosa as in the current study might be one possible reason for higher number mucosal adhesion of *F. varium* DF30 than of *B. vulgatus* DF28. Another possible reason might be the expression of a *Fusobacterium* adhesin A (FadA) homologue by *F. varium* which helps the bacteria to bind to mucosa for colonization [40]. Okamoto et al. (2000) reported that isolates of *Fusobacterium nucleatum* adhered to oral epithelial cells and that sub-inhibitory concentrations of different antimicrobials can decrease or increase the bacterial adhesion properties by alterations in their adhesin protein [41]. To investigate the adhesion properties of *B. vulgatus* and *F. varium* at different concentrations, we used two OD600 = 1 and 0.5 dilutions since at a concentration of OD600 = 0.25, the number of bacterial cells was too diluted for proper assessment.

In the current study, *B. vulgatus* DF28 had a moderate level of hydrophobicity properties (46.1%), which was higher when compared to *F. varium* DF30 (12.6%). Cell surface hydrophobicity is a non-specific interaction between bacteria and host cells and plays an important role in the attachment of bacteria to biological surfaces like intestinal mucosa [34]. Lei et al. (2022) reported a moderate level of hydrophobicity (69%) for *B. vulgatus* Bv46 [42]. In other species of *Bacteroides, however,* the reported hydrophobicity level has been lower. *B. fragilis* strains isolated from dogs’ intestinal tracts showed low cell surface hydrophobicity values (7.7%) [43]. Also, Oyston and Handley (1990) reported low to moderate levels (6.6 to 52.1%) of cell surface hydrophobicity in different *B. fragilis* strains [44]. The low hydrophobicity of *B. fragilis* strains may be due to their rather hydrophilic capsules [43,44]. Having high hydrophobicity properties usually gives the bacterial cells the capacity to generate strong interactions with mucosal cells. The binding might be initially weak and reversible but it may lead to a more specific bacterial adhesion mediated by cell surface proteins [45,46]. In our study, *F. varium* DF30 showed a low level of cell surface hydrophobicity. Okamoto et al. (2000) tested isolates of *F. nucleatum* and reported that they were hydrophilic and lacked an association between hydrophobicity and adhesion on a host’s oral cells [41]. Since the adherence of *F. varium* was higher but the hydrophobicity lower than for *B. vulgatus*, it seems more likely that the adherence of *F. varium* is the consequence of specific bacterial cell surface molecules rather than nonspecific hydrophobicity.

One strategy to modulate gut microbiota is the introduction of gut colonizers with known biological activity, such as beneficial microorganisms, through a simple probiotic supplementation or food fortification [47]. The composition of gut microbiota community must be diverse, balanced, and stable to properly perform its functions. Disturbances in the gut microbiota diversity/richness, e.g., by dysbiosis, can interrupt the mutually beneficial relationship between a host and microbiota and lead to enteropathies [48]. A reduction in the relative abundance of obligatory anaerobes such as *Bacteroides* and *Fusobacterium* has been reported in dogs with CE [6,7]. Inflammation has been found to trigger dysbiosis, and when the latter is present, it can act as a risk factor that may worsen inflammation in individuals who are genetically predisposed [49]. Among those commensal gut microbial species, the *Bacteroides* genus, as an obligatory anaerobe and a primary candidate for next-generation probiotics (NGPs) in humans, has attracted considerable attention due to its relationship with the host [50]. These findings suggest that some specific strains of *B. vulgatus* have the potential to act as a probiotic and to prevent intestinal injury. In mice with dextran sulfate sodium (DSS)-induced colitis, the probiotic cocktail effectively alleviated intestinal inflammation through improving gut microbiota and metabolites, suggesting its great potential to be a novel therapeutic approach for IBD patients in humans [51]. In dogs with IBD, treatment with multi-strain probiotic VSL#3 was compared to prednisolone/metronidazole, and in both groups, a significant reduction in clinical and histological scores, as well as CD3+ T-cell infiltration, was observed on day 90 compared to day 0 [19]. A protective effect of VSL#3 strains was associated with an increase in regulatory T-cell markers (FoxP3+ and TGF-β+) and with normalization of dysbiosis by increases in *Faecalibacterium* spp [19]. In another study, dogs with IBD fed VivoMixx^®^ probiotic showed increased expression of tight junction protein indicating the potential beneficial effects of this probiotic on mucosal homeostasis [20]. Therefore, restoring anaerobic bacteria using probiotics to improve intestinal microbiota composition as well as receiving antioxidant supplements could be an effective treatment in canine chronic intestinal inflammation.

The current study had some limitations. In our study, we used adherence to the colonic mucosa and hydrophobicity as in vitro selection tests to predict the functional properties of *B. vulgatus* and *F. varium* for their possible use as probiotics. However, more in vitro selection tests need to be performed in future studies, such as exposure to bile salts and low pH, competitive exclusion of pathogens, and prokaryotic–eukaryotic co-cultures [52]. In vitro tests are suitable preliminary screening assays to narrow down the number of strains for subsequent in vivo tests to confirm the functional traits of a strain which may lead to health benefits in human or animal subjects [52]. In our study, we incubated the tissue slides with overlaid bacteria overnight at a temperature of +4 °C to protect the biological material from deterioration and to allow the bacteria to bind to mucosa. In in vivo conditions, the temperature is higher and the transit time is shorter; however, the scope of the assay is to measure the adhesion capacities of the bacteria rather than fully mimic a clinical setting. Consequently, it is important to conduct further studies to understand the effects of different incubation times and temperatures on bacterial adhesion properties to paraffin-embedded intestinal tissue slides. Another limitation was that the bacterial adhesion method was not performed under anaerobic conditions, since it was impractical to use them in our study. Future studies should be performed also under anaerobic conditions. Another important limitation was that we examined the adhesion capacity of these bacterial species in the colonic tissues of healthy dogs only. Possible dysbiosis and intestinal inflammatory conditions in dogs with CE might cause the mucosal condition of the intestine to differ from that in healthy dogs. Further research involving intestinal tissue samples from dogs with CE is needed to study also the intestinal adhesion capacities of *B. vulgatus* DF28 and *F. varium* DF30 in CE dogs. Investigating the adhesion properties and hydrophobicity of the two anaerobic bacteria in our study was a preliminary step towards the use of obligatory anaerobes as probiotics; however, there are still important challenges that need to be overcome. These include proper strain characterization and safety assessment, establishing appropriate production technologies, and providing suitable delivery vehicles/formulations to guarantee the supply of sufficient viable cell numbers until the time of consumption.

## 5. Conclusions

In this study, we successfully used paraffin-embedded colonic tissue sections of healthy Beagle dogs to investigate the adhesion properties of Alexa Fluor-labeled *B. vulgatus* DF28 and *F. varium* DF30 representing anaerobic bacterial strains with possible probiotic properties. The study findings revealed that both canine *B. vulgatus* DF28 and *F. varium* DF30 adhered in high numbers to canine colonic mucosa; however, *F. varium* DF30 had a lower hydrophobicity but adhered in higher numbers than *B. vulgatus* DF28. Further studies are needed to determine the efficacy and safety of the strains to be used as potential probiotics, which strains to use, and to reveal the reasons other than hydrophobicity for attachment.

## Figures and Tables

**Table 1:** The bacterial strains used in this study.

	Strain	Origin
*Bacteroides vulgatus*	DF28	Dog feces; isolated at the Microbiome Laboratory, Orion Corporation, Orion Pharma, R&D, Turku, Finland.
*Fusobacterium varium*	DF30	

## Data Availability

Data are contained within the article.
